# Non-destructive Determination of Disintegration Time and Dissolution in Immediate Release Tablets by Terahertz Transmission Measurements

**DOI:** 10.1007/s11095-017-2108-4

**Published:** 2017-02-02

**Authors:** Daniel Markl, Johanna Sauerwein, Daniel J. Goodwin, Sander van den Ban, J. Axel Zeitler

**Affiliations:** 1 Department of Chemical Engineering and Biotechnology, University of Cambridge, Philippa Fawcett Drive, Cambridge, CB3 0AS UK; 2 GSK Research and Development, New Frontiers Science Park, 3rd Avenue, Harlow, CM19 5AW UK; 3 GSK Global Manufacturing and Supply, Priory St, Ware, SG12 0DJ UK

**Keywords:** density, disintegration time, dissolution, granulation, hardness, porosity, refractive index, terahertz

## Abstract

**Purpose:**

The aim of this study was to establish the suitability of terahertz (THz) transmission measurements to accurately measure and predict the critical quality attributes of disintegration time and the amount of active pharmaceutical ingredient (API) dissolved after 15, 20 and 25 min for commercial tablets processed at production scale.

**Methods:**

Samples of 18 batches of biconvex tablets from a production-scale design of experiments study into exploring the design space of a commercial tablet manufacturing process were used. The tablet production involved the process steps of high-shear wet granulation, fluid-bed drying and subsequent compaction. The 18 batches were produced using a 4 factor split plot design to study the effects of process changes on the disintegration time. Non-destructive and contactless terahertz transmission measurements of the whole tablets without prior sample preparation were performed to measure the effective refractive index and absorption coefficient of 6 tablets per batch.

**Results:**

The disintegration time (*R* ^2^ = 0.86) and API dissolved after 15 min (*R* ^2^ = 0.96) linearly correlates with the effective refractive index, *n*_eff_, measured at terahertz frequencies. In contrast, no such correlation could be established from conventional hardness measurements. The magnitude of *n*_eff_ represents the optical density of the sample and thus it reflects both changes in tablet porosity as well as granule density. For the absorption coefficient, *α*_eff_, we observed a better correlation with dissolution after 20 min (*R* ^2^ = 0.96) and a weaker correlation with disintegration (*R* ^2^ = 0.83) compared to *n*_eff_.

**Conclusion:**

The measurements of *n*_eff_ and *α*_eff_ provide promising predictors for the disintegration and dissolution time of tablets. The high penetration power of terahertz radiation makes it possible to sample a significant volume proportion of a tablet without any prior sample preparation. Together with the short measurement time (seconds), the potential to measure content uniformity and the fact that the method requires no chemometric models this technology shows clear promise to be established as a process analyser to non-destructively predict critical quality attributes of tablets.

**Electronic supplementary material:**

The online version of this article (doi:10.1007/s11095-017-2108-4) contains supplementary material, which is available to authorized users.

## INTRODUCTION

Processing of active pharmaceutical ingredients (APIs) into convenient dosage forms for effective drug administration places great demands on excipients, the formulation and the processes used in pharmaceutical production. A variety of excipients, such as diluents, disintegrants, surfactants and lubricants, are mixed to form uniform blends of suitable flowability and compactibility that do not stick to the surfaces of feeder walls, tablet punches or other processing equipment and do not demix during processing [1]. Tablet formulators also have to consider the desired release profile of the dosage form, in particular whether the drug should be released immediately after administration or the drug release should be controlled over a longer period of time. Therefore, the majority of marketed tablets are complex powder compacts containing various different materials which are consolidated by several successive process steps. One of the key processes is powder compaction to form interparticulate bonds, *i.e.*, solid bridges, intermolecular bonds, mechanical interlocking. The strength of these bonds and the arrangement of the particles in the tablet are affected by the material properties (*e.g.*, particle shape, particle size distribution, elasticity/plasticity/brittleness of particles) and the process configuration (*e.g.*, compression speed and force, granulation and drying parameters). Besides the formation of interparticulate bonds the compaction of powder results in a complex pore network within a tablet, which is of central importance for the disintegration and dissolution performance. The pore structure defines the capillary and viscous forces which govern the ingress of gastrointestinal fluids into the tablet upon administration and initiate the disruption of the particle bonds. Such pore structures are typically characterised by the relative void space in the tablet, *i.e.* the porosity, which is the fraction of the volume of voids over the total volume of the tablet. The porosity of a tablet is therefore one of the most important contributors to tablet disintegration and it is directly influenced by the process configuration, such as compaction force and compression speed [2, 3].

Moreover, unfavourable API particle properties, such as their shape or electrostatic properties, may result in the requirement to granulate the API and excipients prior to compaction resulting in denser, larger particles which are more amenable to processing. Granulation thus affects the properties of the particles/granules which are subsequently used for the tablet compaction and ultimately it impacts on the disintegration performance of the final tablet. In particular, the granule density was shown to have a significant effect on the compactibility as well as the performance of the final dosage form [4]. Another aspect in solid processing is whether the disintegrant is added to the formulation before (intra-granular) or after (inter-granular) granulation, as this choice significantly affects the disintegration behaviour of the resulting product [5–8].

It is common practice to use weight, thickness and hardness (breaking force) measurements of tablets following the compaction step as a manufacturing in-process control tool to predict the disintegration performance. Since the tablet breaking force is typically called hardness in the pharmaceutical literature, we will henceforth use both terms interchangeably when referring to the tablet breaking force. Therefore, in the context of this study the tablet hardness is not the resistance of a surface to indentation by a small probe as it is defined in other scientific communities such as materials science. However, weight, thickness and hardness are only indirect descriptors of the critical quality attributes (*i.e.*, porosity and density of individual granules) of complex powder compacts which are required for the accurate prediction of the disintegration performance. Even though the disintegration performance can be mostly inferred from the combination of weight, thickness and hardness measurements, pharmaceutical companies as well as regulatory bodies would prefer a non-destructive direct measure of a large number of tablets providing statistically meaningful insights into the quality of an entire production-scale batch. Such information can only be generated by means of a non-destructive, fast and, most importantly, sensitive method. The low absorption and insignificant scattering losses of terahertz radiation for pharmaceutical formulations result in high penetration power that enable transmission measurements through entire tablets. Together with the non-ionising nature of terahertz radiation as well as the short measurement time (milliseconds) this technology could provide a potential process analytical technology (PAT) tool to non-destructively characterise pharmaceutical tablets, *e.g.*, coating structures, porosity, hardness and density [9].

The analysis of porosity, hardness or density from terahertz measurements is based on its relation to the effective refractive index of the probed sample. In particular, Bawuah *et al.* demonstrated in several studies [10–12] that the effective refractive index can be used to predict the tablet porosity and the intrinsic refractive indices of each tablet component. The authors demonstrated the method by measuring the porosity of thin flat-faced tablets made from pure microcrystalline cellulose (MCC) [10] as well as flat-faced tablets containing MCC and an API [11] before extending the concept to thicker biconvex tablets [12].

The investigations of these relatively simple, direct compacted, MCC formulations form the basis for this study. We applied the same analysis method to investigate tablets containing one API in addition to five excipients. Rather than by direct compaction, all samples were produced by wet granulation, fluid-bed drying and subsequent compaction. The effective refractive index of 18 different batches from a production-scale Design of Experiments (DoE) study were measured by terahertz time-domain spectroscopy (THz-TDS). An average effective refractive index per batch was then related to its respective disintegration time.

## MATERIALS AND METHODS

### Materials and Manufacturing

Details of the formulation and process used to manufacture tablet cores are provided in [13]. Briefly, a micronised, poorly water soluble drug substance was subjected to high shear wet granulation in the presence of lactose monohydrate, microcrystalline cellulose, hypromellose and croscarmellose sodium. The granules were dried in a fluid-bed dryer, screened and blended with extra-granular croscarmellose sodium and magnesium stearate prior to compression. The press was a Fette 2090 tablet press (Fette Compacting GmbH, Schwarzenberg, Germany, fitted with 43 stations). Tablets were compressed to 400 ± 12 mg target weight using 10.5 mm dual radius punches with target tablet breaking force according to Table I. The tablet thickness (*H*) varied depending on the targeted tablet breaking force and the tableting speed was 140,000 tablets per hour.

**Table I:** List of production scale experiments selected from a 4 factor split - plot DoE

Batch ID	Water amount	Water addition rate	Wet massing time	Median granule size	Tablet thickness	Tablet wall height	Tablet breaking force	Solid fraction	Granule density	Disintegration time
	wt%	g/min/kg	min	μm	mm	mm	kp		g/cm^3^	s
B01	27	14	2	99	5.27	1.98	7.5	0.83	0.60	274
B02	27	14	2	99	5.10	1.84	10.5	0.87	0.60	412
B03	27	24	2	110	5.31	2.04	7.5	0.82	0.59	437
B04	27	14	6	119	5.21	1.91	7.0	0.85	0.63	470
B05	27	24	2	110	5.06	1.76	11.3	0.89	0.59	513
B06	32	14	2	119	5.15	1.86	7.0	0.86	0.64	602
B07	30	19	4	121	4.97	1.64	9.4	0.92	0.63	627
B08	27	14	6	119	4.94	1.67	10.6	0.91	0.63	646
B09	32	24	2	131	5.13	1.84	6.2	0.87	0.64	656
B10	32	24	6	110	4.85	1.62	5.5	0.93	0.69	704
B11	27	24	2	110	4.86	1.56	15.5	0.94	0.59	720
B12	27	14	2	99	4.84	1.56	15.3	0.94	0.60	721
B13	32	24	6	110	4.87	1.63	6.5	0.93	0.69	726
B14	32	24	2	131	4.82	1.54	10.1	0.95	0.64	794
B15	32	24	6	110	4.86	1.62	6.4	0.93	0.69	811
B16	32	14	2	119	4.88	1.60	10.9	0.93	0.64	832
B17	32	24	2	131	4.67	1.39	13.5	0.99	0.64	874
B18	27	14	6	119	4.73	1.44	15.5	0.98	0.64	898

Three factors were changed at three levels for the high shear wet granulation step (water amount: 27, 30, 32 wt%; water addition rate: 14, 19, 24 g/min/kg according to dry weight; and wetting time: 2, 4, 6 min) and one factor was changed for the tablet press (tablet hardness/breaking force: 7, 11, 15 kp). The tablet breaking force represents the average value from the measurement of 20–45 tablets from the entire batch, which were sampled during compression at fixed time intervals of 15 min for all batches except for B07 which was sampled at 30 min time intervals. The granule density was computed from the fill depth, tablet weight and tablet dimensions: *ρ*_*g*_ = (*m*_tablet_)/(0.25*H*_fill_ *πD* ^2^ + *V*_cup_), where *m*_tablet_ is the tablet mass, *H*_fill_ the fill depth, *D* the tablet diameter and *V*_cup_ the tablet cup volume.

The batches are numbered with respect to their disintegration time (from low to high).

The tablet batches were generated from a split-plot DoE across three granulation factors (water amount, wet massing time and water addition rate) and one compression factor (tablet breaking force). Only a subset of the produced batches for the split-plot DoE was selected for this study as indicated in Table I. For all batches except B07, the granulation and blending batch size was 163 kg, with each blend split into 3 sub-lots of 50 kg for compression at three different tablet hardness levels (the excess was not used). For B07, two 163 kg granulation batches were combined at the blending stage and the whole blend was compressed at a single tablet hardness.

The tablet weight, thickness and breaking force were measured using a Pharmatron 10× automated tablet system (Dr. Schleuniger, Thun, Switzerland). Disintegration and dissolution were determined following the pharmacopeial methods (the US and European pharmacopeias). For disintegration testing an Erweka ZT3 (Erweka GmbH, Heusenstamm, Germany) was used and the disintegration time was defined as the time at which the last tablet disintegrated out of a set of 6 tablets. The disintegration test was performed once per batch and hence no standard deviation was measured. Dissolution testing was carried out using the Sotax AT7 Smart dissolution bath (Sotax AG, Aesch, Switzerland) together with the Waters Alliance 2695D separation module HPLC system with sample transfer module and 2487 absorbance detector (Waters Ltd., Elstree, U.K.).

### Terahertz Time-Domain Measurements

**Fig. 1:**
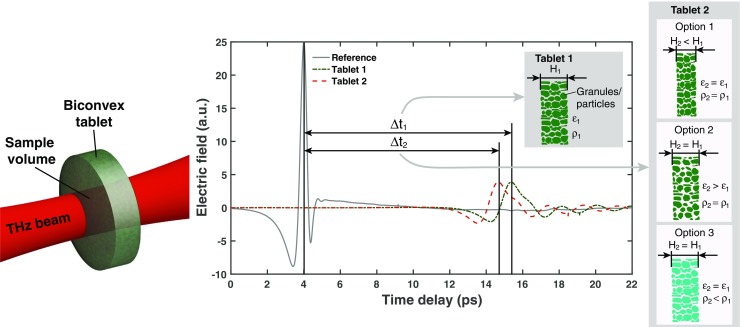
Schematic of a terahertz transmission measurement of the biconvex tablets and terahertz time-domain waveforms from a reference, a tablet from batch B18 (Tablet 1) and a tablet from batch B07 (Tablet 2). The difference in phase shift between Tablet 1 and Tablet 2 is either due to a thinner tablet (*H*, option 1), a larger porosity (*ε*, option 2), a lower density of the granules (*p*, option 3) or due to a combination of all three options. The subscripts of the time delay Δ*t*, the tablet thickness *H*, the porosity *ε*, and the the density *p* assign each variable to either Tablet 1 or Tablet 2.

Terahertz time-domain measurements of 6 tablets per batch (108 tablets in total) were acquired using a TeraPulse 4000 (TeraView Ltd., Cambridge, UK) in combination with a transmission module at an instrument resolution of 0.01 ps over a total range of 150 ps for each time-domain waveform. No sample preparation was required and the measurements represent the transmitted terahertz waveforms acquired at the centre of each respective tablet in axial direction (Fig. 1). The transmission module was purged with dry nitrogen gas throughout the measurement. Co-averaging of 60 measurements was performed in order to reduce the noise variance. Each resulting terahertz waveform is composed of 15,000 data points and the measurement of each co-averaged waveform took a total of 1.5 min.

By comparing the sample waveforms with a reference waveform (waveform with no sample present in the measurement chamber) the pulse delay time Δ*t* was determined for each sample (see Fig. 1).

In general, Δ*t* of a terahertz pulse propagating through a sample depends on the effective refractive index, *n*_eff_, as well as the tablet thickness. The value of *n*_eff_ of a tablet can be readily calculated from the pulse delay time as follows:

1 $$ {n}_{\mathrm{eff}}=\frac{c\varDelta t}{H}+1, $$

where *H* is the thickness of the tablet and *c* is the speed of light in vacuum. Therefore, by measuring the time delay difference of different samples (*e.g.*, Δ*t*_1_ and Δ*t*_2_ in Fig. 1) the value of *n*_eff_ is calculated for each sample. The tablet thickness was measured for every single tablet using a micrometer (Expert Metric External Micrometer 0–25 mm, Draper Expert, Hampshire, UK). Variations in *n*_eff_ of different tablets with the same formulation are soly related to changes in the tablet microstructure, *i.e.* their porosity and density.

Besides the straightforward time-domain analysis the terahertz data can also be analysed in the frequency-domain. Since the electric field of the transmitted terahertz pulse is measured directly, we can extract both the frequency-dependent absorption coefficient and effective refractive index of a tablet from a simple terahertz transmission measurement without any mathematical processing by means of the Kramers-Kronig relationship. Applying a discrete Fourier transform on the time-domain waveforms (reference and sample) yields their respective spectra. The sample spectrum is then normalised by the reference spectrum resulting in the complex-valued transmission spectrum. By assuming the tablet as a homogeneous medium, the frequency-dependent effective refractive index and the effective absorption coefficient can be determined by combining the transmission spectrum, Beer-Lambert’s law and the measured tablet thickness. More details about the extraction of optical constants from terahertz measurements is provided by Zeitler [9].

## RESULTS AND DISCUSSION

**Fig. 2:**
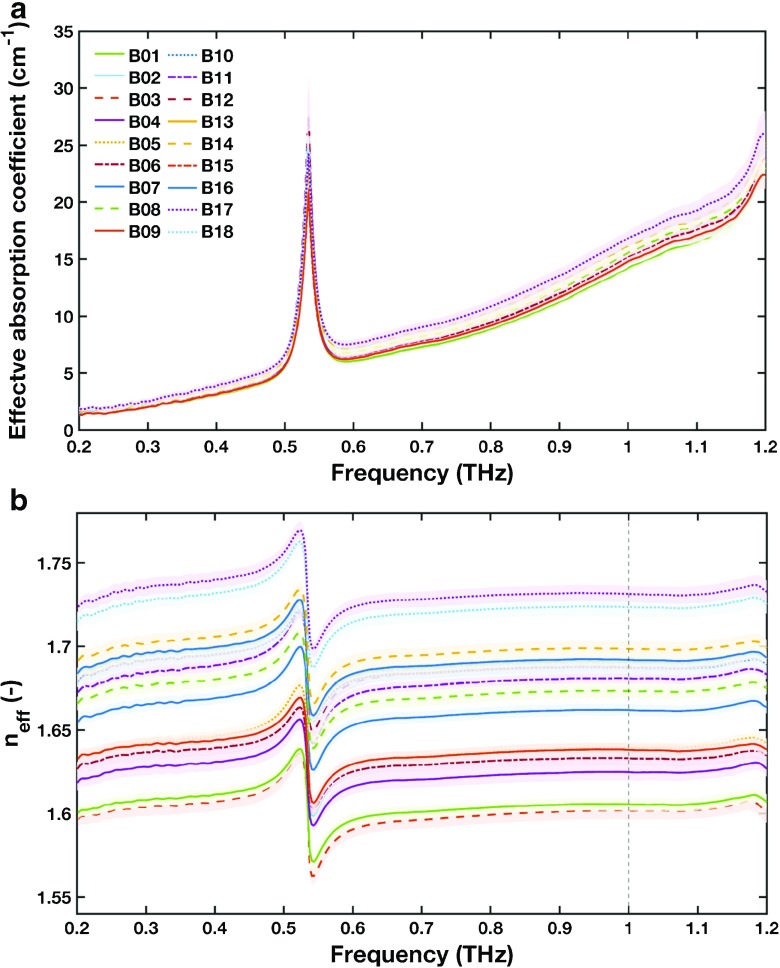
Frequency dependent (**a**) effective absorption coefficient and (**b**) effective refractive index of all batches. The lines represent the average values and the shaded areas the standard deviations of each batch. The dashed vertical line in b) at the frequency of 1 THz highlights the data used for further interpretations of *n*_eff_.

The frequency-dependent analysis of the terahertz waveforms provides the effective absorption coefficient (Fig. 2a) and the effective refractive index (Fig. 2b). A single peak is observed in the effective absorption coefficient at a frequency of 0.53 THz, indicating the presence of phonon vibrations due to one of the crystalline constituents in the sample. As expected, the absorption feature is accompanied by relatively strong dispersion at this frequency [14]. Both the spectrally resolved absorption coefficient and the refractive index reflect the changes of the physical properties of the tablets. A plateau-like behaviour of the refractive index in the range 0.6–1.11 THz shows that there is no dispersion of the terahertz wave at these frequencies. The spectral windows where absorption is relatively weak can further be used to analyse the relationship of the effective refractive index and other properties of the powder compact, which will be the focus of future studies.

**Fig. 3:**
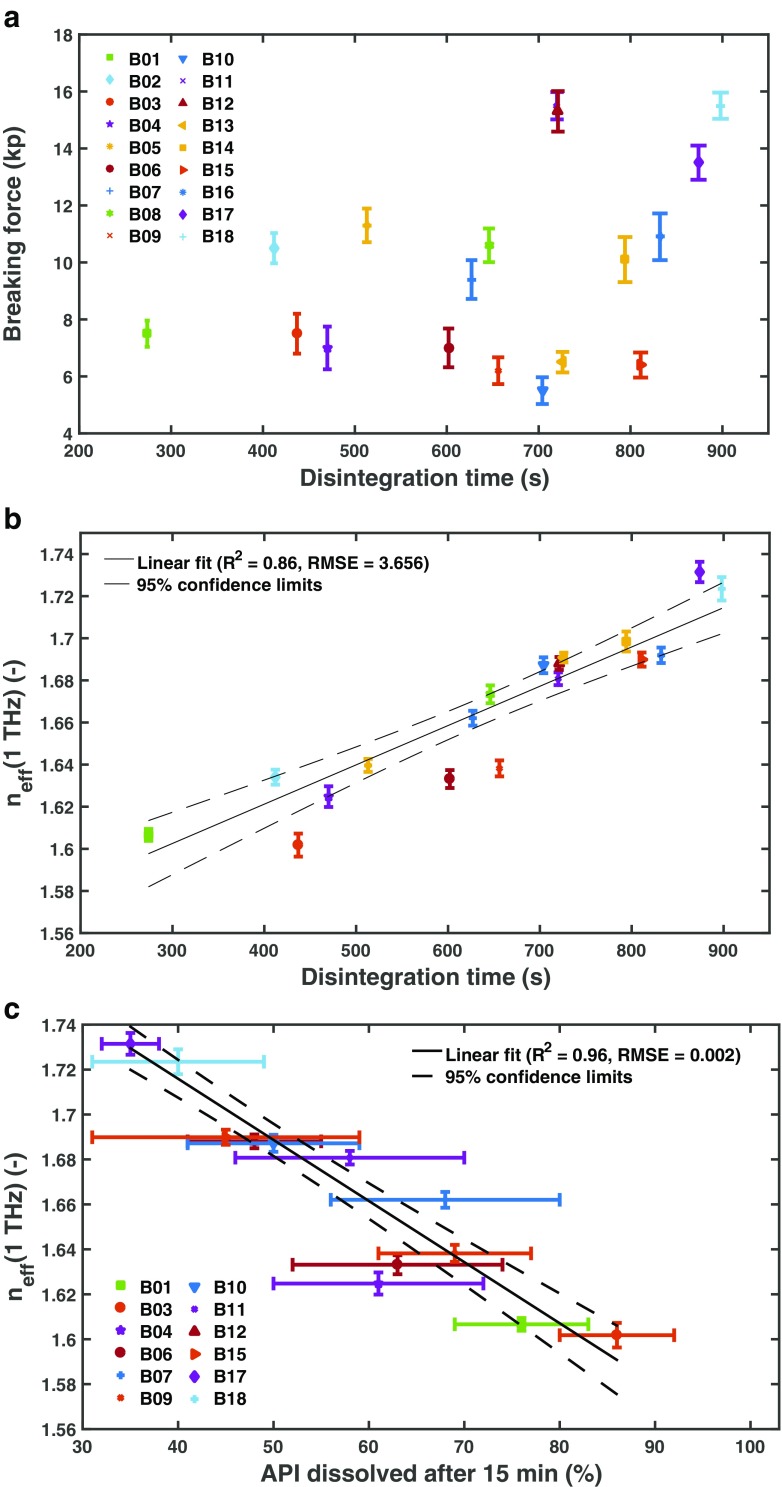
Average (**a**) breaking force and (**b**) effective refractive index, *n*_eff_(1 THz), as a function of the disintegration time. The effective refractive index was evaluated for each tablet at a frequency of 1 THz (see **Fig.** 2b). The average and standard deviation of the effective refractive index was calculated from 6 tablets per batch. c) The effective refractive index, *n*_eff_(1 THz), as a function of the API dissolved after 15 min. Dissolution testing was only performed for a subset of all batches as indicated in the figure legend. The error bars show the standard deviation of 12 independent samples and a weighted liner fit was perfromed using a weighting of 1/(*σ*(*x*)^2^ + *σ*(*n*_eff_)^2^), where *σ* is the standard deviation and *x* is the amount of API dissolved.

In order to analyse the relation of the effective refractive index and the disintegration time in more detail, we chose the effective refractive index at a frequency of 1 THz (Fig. 3b). The small standard deviation of the refractive index at this frequency indicates a high consistency within each batch as well as a good reproducibility of the terahertz measurement. The choice of frequency is flexible as long as a frequency is chosen at which no significant dispersion due to a spectral feature occurs. From a practical point of view it is favourable to select the frequency close to the spectral range where the measurement instrument exhibits its maximum dynamic range and hence the signal-to-noise characteristics are optimised.

Figure 3a illustrates the breaking force as a function of disintegration time. The breaking force, or the tablet hardness that is derived from the breaking force, is typically used for controlling the quality of the tablets.

A proper comparison of tablets with different shapes would require to calculate tensile strength as well as to normalise the disintegration time by the surface area of each tablet. The results of tensile strength and normalised disintegration time are presented in the supporting information of this manuscript. As pointed out in the supporting information, the relationship is very similar to that of the breaking force and the disintegration time (Fig. 3a). It is apparent that there is no clear correlation between the breaking force and the disintegration time as well as between tensile strength and normalised disintegration force. In contrast, there is a clear linear correlation between the disintegration time and the effective refractive index (Fig. 3b, *R* ^2^ = 0.86). The breaking force primarily reflects changes in the compaction process related to the bonding structure and bonding mechanisms of the tablets [3, 15] as well as porosity [16], whereas it is not significantly impacted by variations of granule properties such as their density. However, *n*_eff_ accounts for porosity as well as density changes (see supporting information for more details) and thus represents in a more sensitive descriptor, and in turn critical quality attribute, of such tablets in contrast to the breaking force (*i.e.*, hardness test). We additionally observe a linear correlation between *n*_eff_ and the amount of API dissolved after 15 min (Fig. 3c, *R* ^2^ = 0.96), 20 and 25 min (dissolved API > 82% for all batches). The dissolution results from 20 min (*R* ^2^ = 0.88) and 25 min (*R* ^2^ = 0.79) are presented in the supporting information. The pore structure and density impact the disintegration performance in particular and thus strongly influence the early dissolution performance. This is reflected by the *R* ^2^, which decreases with increasing dissolution time. Comparing Figs. 3b (*n*_eff_ vs disintegration time) and c (*n*_eff_ vs API dissolved after 15 min) reveals that whilst some batches are outside of the confidence interval (B03, B06, B09, B10 and B17) in Fig. 3b they only marginally deviate from the fitted line in Fig. 3c (*i.e.*, B03, B09, B10 and B17). This apparent discrepancy in the predictive power of the method can easily be explained by the rather limited accuracy of the disintegration testing approach where measurements of multiple samples are aggregated and the endpoint determination of the test is rather ill defined. The magnitude of the error in the amount of API dissolved shown in Fig. 3c highlights the significant variability in the dissolution behaviour per batch (relative standard deviation of 7–31% were measured at 15 min dissolution time). In contrast, the data for disintegration appears to show much less variation but it is important to reiterate that the disintegration time merely represents the time at which the last of six tablets disintegrated. It provides no information about the variation in disintegration time between the six individual tablets. Further research into alternative disintegration testing methods are necessary to better understand the limitation of the current methods.

It is well-known that the effective refractive index can be used as a measure of porosity [11] and density [17]. An increase in porosity results in an optically less dense medium due to the negligible absorption of air as well as its low refractive index (*n*_air_ = 1) compared to the API and excipients. A less dense medium can also be caused by a lower density of the particles or granules. Since the density and the porosity of the powder compact are closely related to the mechanical strength of the tablet, the terahertz technique is particularly powerful in providing a measure for the disintegration performance. This is conceptually different to hardness measurements by means of near-infrared [18–20] or Raman [21] spectroscopies, where the surface hardness is correlated to scattering losses. Such scattering losses depend on the surface roughness and the size of pores which are of length scales similar to the wavelength of the light used by these techniques (typically 500–1100 nm). In contrast, terahertz radiation corresponds to a wavelength range from 100 μm − 3 mm. Consequently, the effect of structural variations of the powder compacts on the terahertz measurements is not primarily due to scattering but rather a change in optical density of the probed medium.

In general, the effect of the different process configurations on *n*_eff_ is a combination of the changes in density of the granules and in the pore structure of the tablet. An increase of the porosity of the tablet and/or a decrease of the granule density thus causes a decrease in *n*_eff_. It was previously shown that a lower granule density is related to a lower moisture content in the granules [4, 22].

The amount of water applied during the granulation is listed in Table I for each batch. Our results highlight that it is not only the amount of liquid that influences the disintegration time but also the wet massing time. It was also shown by van den Ban and Goodwin [13] that the water addition rate had not a statistically significant impact on granule density. Tablets from batches with similar breaking forces disintegrate faster when less water was applied during granulation and when the wet massing time was shorter. The fastest disintegration was thus observed for batches B01 and B02. Of these two batches B01 disintegrated even quicker than B02 given the lower breaking force and thus larger tablet porosity. This behaviour is in good agreement with the fact that fast absorption of a fluid was observed for powder compacts that exhibit a high porosity [23, 24]. While fast fluid penetration does not directly result in the building up of the pressure, which is necessary to break the interparticulate bonds, it is a prerequisite to initiate other mechanisms like swelling of the disintegrant [25, 26]. In contrast, we observed the slowest disintegration in batch B18, which was processed with a low amount of water but which was exposed to a long wet massing time and exhibited the highest breaking force of all the batches investigated.

**Fig. 4:**
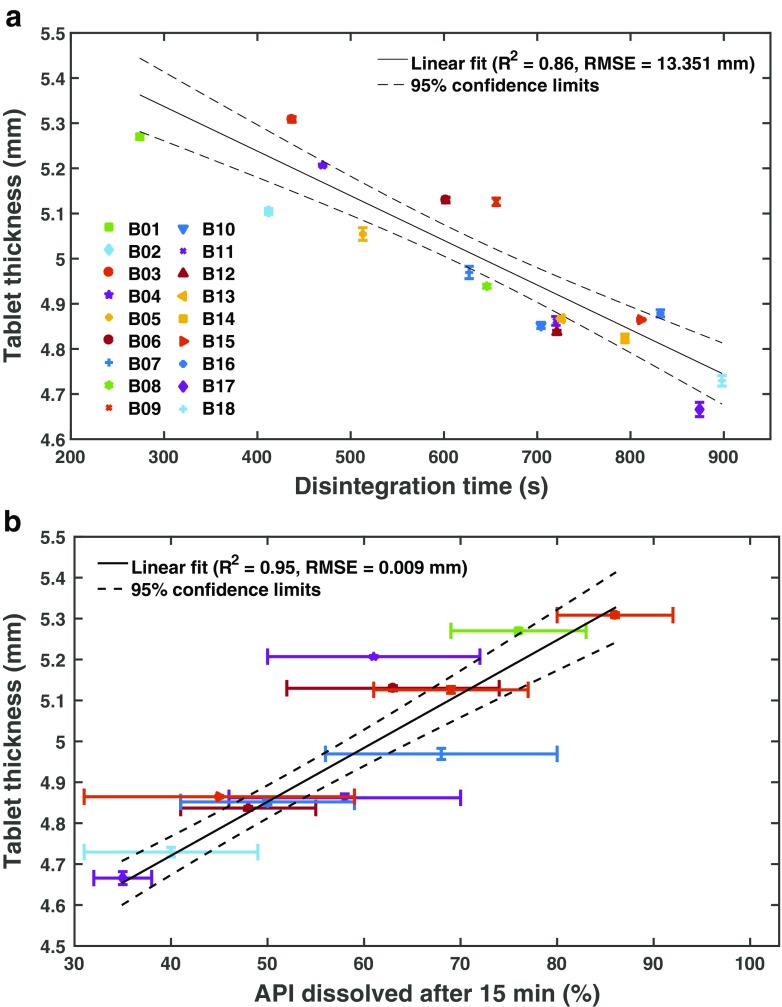
Average thickness as a function of disintegration time (**a**) and API dissolved after 15 min (**b**). The average and standard deviation of the thickness was calculated from 6 tablets per batch. Dissolution testing was only performed for a subset of all batches as indicated in the figure legend. The *error bars* show the standard deviation of 12 independent samples and a weighted liner fit was perfromed using a weighting of 1/(*σ*(*x*)^2^ + *σ*(*n*_eff_)^2^), where *σ* is the standard deviation and *x* is the amount of API dissolved.

**Fig. 5:**
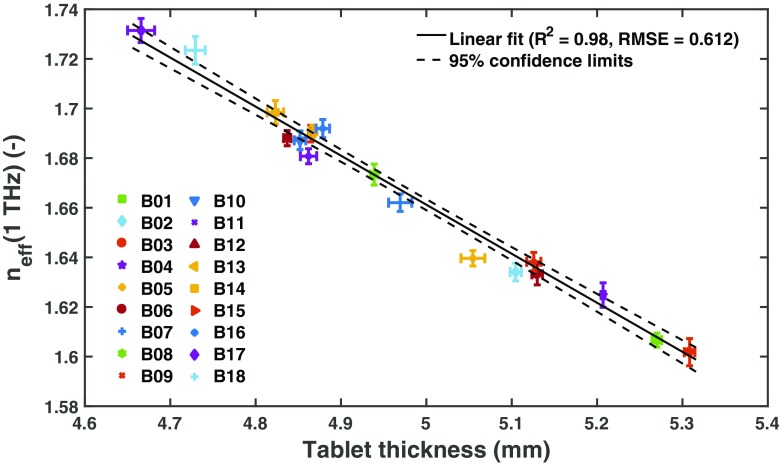
Correlation between ***n***_**eff**_ and tablet thickness.

In addition to the terahertz measurements we also observe a good correlation between the disintegration time and dissolved API after 15 min with the tablet thickness (Fig. 4. This correlation is not significantly worse compared to using *n*_eff_ for predicting the dissolution behaviour, which is not surprising given that for this set of samples the thickness and the refractive index correlate almost perfectly (Fig. 5). However, it is very important to see these results in the context of the pronounced variability in dissolution and disintegration discussed above. The relative variation in *n*_eff_ is higher (0.23 ± 0.06%) compared to that of the thickness (0.16 ± 0.08%). It will be critical to examine in future whether this variability reflects a better sensitivity to the disintegration process by correlating both the thickness and *n*_eff_ against the dissolution results measured on the exact sample specimens rather than an average batch quantity.

Since tablet thickness is a shape parameter, it is sensitive to mass and volume variations of each sample. Mass and volume of the tablet are impacted by cumulative variations in particle flow, inherent powder density, particle size, differences upon filling the die as well as variations in granule density. On the contrary, *n*_eff_ is a bulk property which is only affected by variations in granule density and overall porosity given that the refractive index is derived from the pulse time delay Δ*t* by dividing with the thickness of the actual sample specimen. In the well controlled tableting experiments described in this study these particle properties are kept in tight check which causes the excellent correlation between tablet thickness and refractive index. The terahertz method should in principle be more sensitive to detecting deviations from the uniformity of the particle properties as these are likely to have far higher impact on *n*_eff_ compared to the tablet thickness. From a PAT perspective the terahertz method has the additional advantage that it is not reliant on performing two independent measurements, *i.e.* measuring thickness and weight of a sample, but that any deviations are picked up in a single non-contact measurement. Besides these advantages the terahertz method is capable of detecting not only minute changes in the physical structure of the tablet matrix but also changes in the chemical composition. If inhomogeneities in the powder or granule mixture develop during the process run this can be picked up not only by the subtle changes in relative refractive index but directly in the absorption data as spectral changes [9]. The method is in principle capable to ensure content uniformity as well as provide predictions for the tablet disintegration. However, as outlined above, within the inherent limitations of the disintegration testing and the limitations of using dissolution testing results based on cumulative batch data, both tablet thickness and *n*_eff_ show good correlations for the tablets under investigation with the disintegration time and the API dissolved after 15 min.

Besides the analysis of the refractive index, *n*_eff_, it is also possible investigate the correlation between the disintegration and dissolution with the absorption and scattering losses detected by the terahertz signal. Rather than resolving the solid fraction of the tablet matrix this analysis is sensitive to the physical domain size in the microstructure of the tablet matrix. Each tablet can be considered as a porous medium that is comprised of relatively homogenous particles that are tightly packed together and are either in direct contact with one another or separated by the pore space. Given that there is a significant difference in refractive index between the compacted granules (1.6 < *n*_eff_ < 1.7) and air (*n* = 1) and that the domain size of the microstructure approaches length-scales of the order of the wavelength (the bandwidth of 0.2 to 1.2 THz corresponds to wavelengths of 1.5 to 0.25 mm) scattering losses will occur upon the propagation of the terahertz pulse through the tablet matrix. The variation in the effective absorption data shown in Fig. 2 is the direct result of the scattering losses as all samples were prepared using the same mass of sample material and the absorption is normalised by the sample thickness.

Shen, Taday and Pepper previously showed that the scattering losses in absorbing media can be described empirically using a power law [27]:

2 $$ {\alpha}_{\mathrm{eff}}(v)= B{v}^A, $$

where *α*_eff_(*ν*) is the effective absorption coefficient (with the frequency *v*) and parameters *A* and *B* are fitting parameters, with *A* being constant and *B* expected to increase with the particle (or here domain) size as discussed by Shen, Taday and Pepper [27]. Therefore, we calculated the fit parameters *A* and *B* of Eq. 2 using the measured *α*_eff_(*ν*) in the frequency range of 0.65 < *v* < 1.2 THz.

**Fig. 6:**
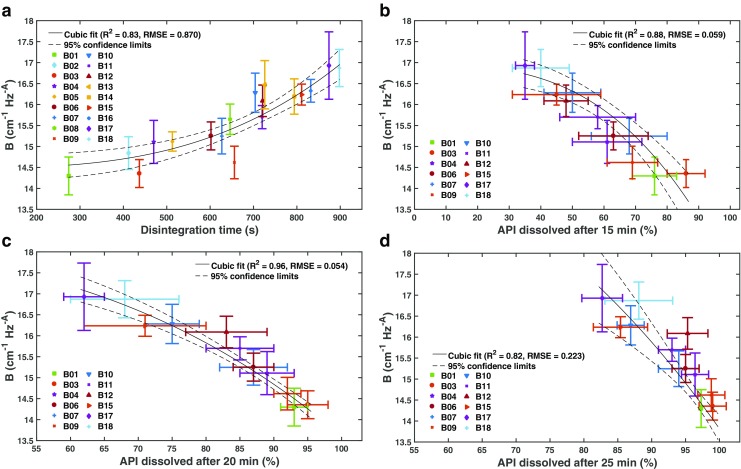
Correlation between the domain size descriptor *B* extracted from the scattering analysis (see Eq. **2**) and the (**a**) disintegration time and the dissolved API after (**b**) 15 min, (**c**) 20 min and (**d**) 25 min. The data was fitted by a cubic function ***B*** = ***β***_1_ + ***β***_2_ ***x*** ^3^ with *x* as the API dissolved after the given time and with the fitting parameters ***β***_1_ and ***β***_2_.

The data show an excellent correlation between parameter *B* and the amount of dissolved API after 15 and 20 min (Fig. 6). The best fit between *B* and the dissolved API could be established by a cubic function (*B* ∼ *x* ^3^ with *x* as the amount of API dissolved). The correlation of *B* with the disintegration time is slightly weaker (Fig. 6a). A poorer correlation with disintegration is intuitive in that the disintegration process, *i.e.* the rapid breaking apart of the granules prior to dissolution, is limited by the liquid penetration into the porous medium which is critically dependent on the porosity of the sample (hence the better correlation with *n*_eff_). In contrast, the ‘domain size’ that leads to the scattering losses reflects the initial size of (agglomerated) particles that form immediately following the disintegration of the dosage form. It is well established that smaller particles have a higher intrinsic dissolution rate and hence it is not surprising to see a relatively strong correlation between the scattering parameter and the dissolution data in the early phase of dissolution of this API (BCS class II), in particular given that all tablets are fully disintegrated at 15 min. The correlation with the dissolution data at 25 min is starting to tail off as for most batches the API is fully dissolved at this point. It is important to highlight that there was relatively little variation in granule size prior to compaction (100 − 130 *μ*m) but that the scattering domain size reflects the structure that forms within the powder compact, and which we assume, is more representative to the size of the particles of the disintegrated tablet. The process configurations of the granulation and the compaction thus clearly impacted the domain size of the tablets causing this variation of the parameter *B* as well as in the dissolution behaviour between the different batches.

## CONCLUSION

This study demonstrates the potential of terahertz spectroscopy as an important process monitoring or quality control tool for pharmaceutical tablets prepared by wet granulation and compaction. The method has the advantage that it is non-destructive and contactless. Due to the fact that the effective refractive index reflects density and porosity changes of the granules as well as of the entire tablet, it provides an excellent predictor for the tablet disintegration performance. Furthermore, we found that the effective absorption coefficient, that is derived from the same measurement, is an independent metric reflecting the internal structural domain size which affects the immediate dissolution rate.

Given the potential for automated non-destructive measurements, terahertz technology might be especially advantageous where occupational health categorisation 4 (OHC4) or OHC5 compounds that require high containment are being used in order to prevent the handling of the compounds by the workers. Moreover, the number of measured tablets could be drastically increased, when using this technology as an at-line or on-line tool, to demonstrate uniformity of the product. Further development of the terahertz technology and the processing procedures are, however, necessary in order to transfer the presented concept to sufficiently robust at-line or on-line control of the disintegration/dissolution performance.

## Electronic supplementary material

Below is the link to the electronic supplementary material.

ESM 1 (PDF 1051 kb)

## ABBREVIATIONS

*α*_eff_: Effective absorption coefficient
*n*_eff_: Effective refractive index
API: Active pharmaceutical ingredient
DoE: Design of experiments
MCC: Microcrystalline cellulose
PAT: Process analytical technology
THz-TDS: Terahertz time-domain sprectroscopy
*v*: Frequency
*x*: API concentration
