# Supplementary material for: Non-destructive Determination of Disintegration Time and Dissolution in Immediate Release Tablets by Terahertz Transmission Measurements
Source: Pharm Res. 2017 Feb 2;34(5):1012–22. doi: 10.1007/s11095-017-2108-4 (PMC5382185; doi:10.1007/s11095-017-2108-4)
Supplement: Supplementary file 1 — (PDF 1051 kb) [file 11095_2017_2108_MOESM1_ESM.pdf]

Pharmaceutical Research

SUPPLEMENTARY INFORMATION

Evaluating the Relation between Disintegration Time and Effective Refractive Index From Terahertz Transmission Measurements

Daniel Markl[1] · Johanna Sauerwein[1] · Daniel J Goodwin[2] · Sander van den Ban[3] · J Axel Zeitler[1]

December 22, 2016

ABSTRACT

In this supplementary section we provide results about the tensile strength, effective refractive index, n_{eff}, and solid fraction as a function of the disintegration time normalised by the surface area of the tablet. The results emphasise the dependence of n_{eff} on the solid fraction (1 - porosity) and highlight that the disintegration performance is impacted by the porosity and the granule density.

1 TENSILE STRENGTH AND NORMALISED DISINTEGRATION TIME

It is common practice in the pharmaceutical industry to relate the breaking force to the disintegration time. Since the breaking force is impacted by the tablet shape, we would need to account for the varying tablet thicknesses in order to properly compare the different batches. This can be considered by calculating the tensile strength [1]:

$$\delta_s = \frac{10P}{\pi D^2 \left(2.84\frac{H}{D} - 0.126\frac{H}{W} + 3.15\frac{W}{D} + 0.01\right)}. \quad (1)$$

D, H and W are the tablet diameter, tablet thickness and wall height (of the tablet band), respectively. P is the fracture loading and can be calculated from the breaking force following the definition that one kilopond (kp) is equal to 9.81 N. Furthermore, it is well known that the disintegration time depends on the surface area of the tablet. A varying tablet thickness also changes the surface area, which should be considered when comparing the disintegration times of the different batches. Therefore, the disintegration time was normalised by the surface area of the tablet assuming isotropic surface behaviour during disintegration.

Figure 1 illustrates the dependence of the tensile strength and n_{eff} on the normalised disintegration time. As expected, the breaking force and n_{eff} vs disintegration time ($R^2 = 0.86$) as illustrated in Figure 3 in the main manuscript have a similar trend to the data shown in Figure 1 ($R^2 = 0.88$). The small thickness variations between the batches thus have only a minor effect on the tensile strength and the normalised disintegration time.

2 SOLID FRACTION

The solid fraction (1 - porosity) of a tablet impacts the effective refractive index of a tablet [2,3], which can be clearly observed in Figure 2b and is indicated by the

✉ J A Zeitler
jaz22@cam.ac.uk

[1] Department of Chemical Engineering and Biotechnology, University of Cambridge, West Cambridge Site, Philippa Fawcett, Drive Cambridge, CB3 0AS, UK

[2] GSK Research and Development, New Frontiers Science Park, 3rd Avenue, Harlow, CM19 5AW, UK

[3] GSK Global Manufacturing and Supply, Priory St, Ware. SG12 0DJ, UK

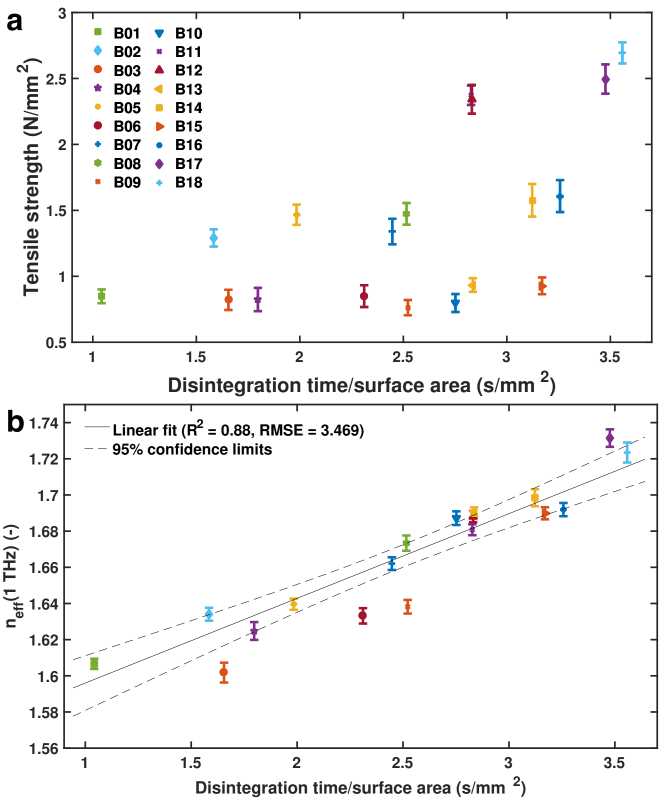

Fig. 1 Average a) tensile strength and b) the effective refractive index, n_{eff}, as a function of the normalised disintegration time.

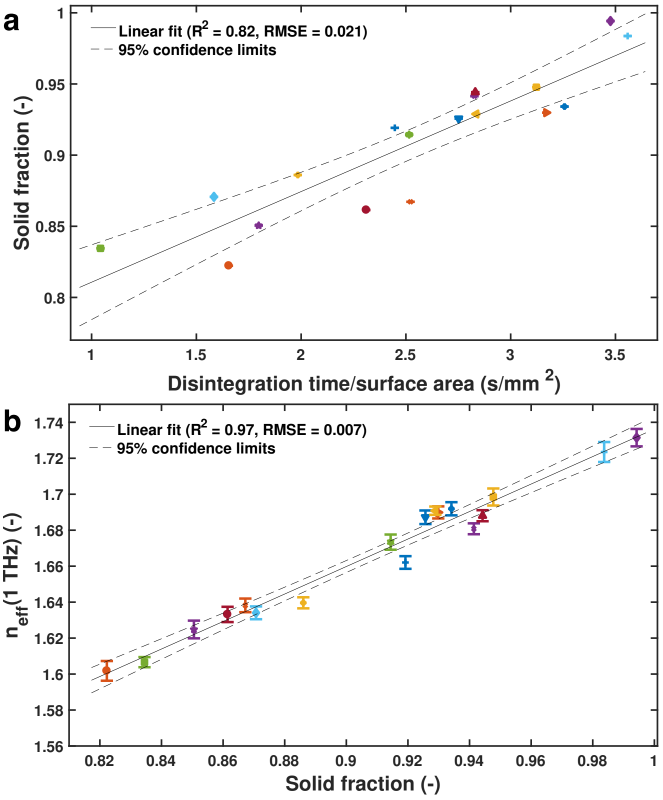

Fig. 2 Average a) solid fraction depending on the normalised disintegration time and b) the effective refractive index, n_{eff}, as a function of the solid fraction.

high R^2 of 0.97. Comparing the solid fraction and the normalised disintegration time (Figure 2) emphasises that the porosity strongly influences the disintegration time. It further highlights that the correlation between the solid fraction and the normalised disintegration time ($R^2 = 0.82$) is smaller than that between n_{eff} and the normalised disintegration time ($R^2 = 0.88$). This indicates that the granule density captured by the terahertz measurements influences the disintegration performance.

3 DISSOLUTION RESULTS

Figure 3 illustrates n_{eff} as a function of the API dissolved after 20 and 25 min. As discussed in the main text, R^2 decreases with increasing dissolution time.

References

1. van den Ban S, Goodwin DJ. The Impact of Granule In-Die Density on Tabletting and Pharmaceutical Product Performance. Pharmaceutical Research. submitted;.
2. Bawuah P, Pierotic Mendia A, Silfsten P, Pääkkönen P, Ervasti T, Ketolainen J, et al. Detection of porosity of pharmaceutical compacts by terahertz radiation transmission and light reflection measurement techniques. International Journal of Pharmaceutics. 2014;465(1-2):70–76.
3. Bawuah P, Tan N, Tweneboah SNA, Ervasti T, Axel Zeitler J, Ketolainen J, et al. Terahertz study on porosity and mass fraction of active pharmaceutical ingredient of pharmaceutical tablets. European Journal of Pharmaceutics and Biopharmaceutics. 2016;105:122–133.

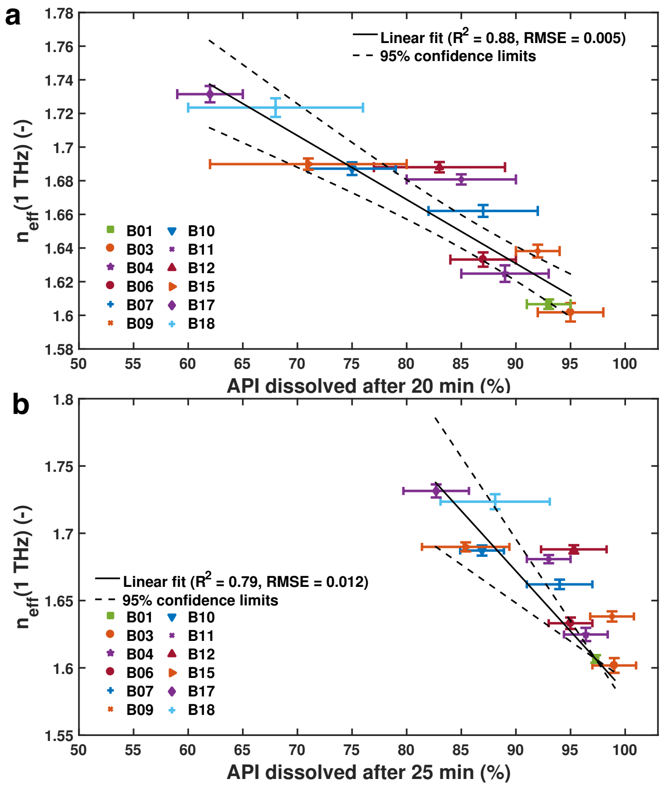

Fig. 3 The effective refractive index, n_{eff}, as a function of the amount of API dissolved after (a) 20 and (b) 25 min.
